# Effects of age, sex and arm on the precision of arm position sense—left-arm superiority in healthy right-handers

**DOI:** 10.3389/fnhum.2013.00915

**Published:** 2013-12-24

**Authors:** Lena Schmidt, Lena Depper, Georg Kerkhoff

**Affiliations:** ^1^Department of Psychology, Clinical Neuropsychology Unit and Outpatient Service, Saarland UniversitySaarbruecken, Germany; ^2^International Research Training Group 1457 “Adaptive Minds”Saarbruecken, Germany

**Keywords:** proprioception, position sense, body, aging, sex, assessment, stroke

## Abstract

Position sense is an important proprioceptive ability. Disorders of arm position sense (APS) often occur after unilateral stroke, and are associated with a negative functional outcome. In the present study we assessed horizontal APS by measuring angular deviations from a visually defined target separately for each arm in a large group of healthy subjects. We analyzed the accuracy and instability of horizontal APS as a function of age, sex and arm. Subjects were required to specify verbally the position of their unseen arm on a 0-90° circuit by comparing the current position with the target position indicated by a LED lamp, while the arm was passively moved by the examiner. Eighty-seven healthy subjects participated in the study, ranging from 20 to 77 years, subdivided into three age groups. The results revealed that APS was not a function of age or sex, but was significantly better in the non-dominant (left) arm in absolute errors (AE) but not in constant errors (CE) across all age groups of right-handed healthy subjects. This indicates a right-hemisphere superiority for left APS in right-handers and neatly fits to the more frequent and more severe left-sided body-related deficits in patients with unilateral stroke (i.e. impaired APS in left spatial neglect, somatoparaphrenia) or in individuals with abnormalities of the right cerebral hemisphere. These clinical issues will be discussed.

## Introduction

Proprioception is defined as the sense of position and movement of the limbs without information coming from the visual system (Fuentes and Bastian, 2010). Proprioceptive abilities are essential for orientation and moving in space and engaging with the environment. They are the basis for goal-directed movements of the limbs as well as for locating our limbs without looking and therefore important for nearly all daily life activities (Carey et al., 1996). This includes functions like the control of aiming accuracy, performance of movement sequences, reaching and tracking movements like grasping and manipulating objects as well as the control and correction of ongoing movements. Impairments in proprioception most likely affect any of those abilities and these deficits are frequent, occurring in some 34-64% of stroke patients (Connell, 2008). Proprioceptive deficits cause difficulties and insecurity in many activities of daily living, can compromise personal safety (Carey et al., 1996) and lead to postural instability when e.g. limbs are used to compensate for balance disturbances (Adamo et al., 2007). There is also a relationship between deficits in proprioception in the elderly and sensorimotor dysfunctions, e.g. postural control or balance, and activities of daily living [for a review, see Goble et al. (2009a)].

## Clinical significance of proprioceptive loss

Loss of limb position occurs in one third to half of stroke patients (Shah, 1978; Smith et al., 1983; Carey, 1995) and mostly affects the contralesional side of the body but can also affect the ipsilesional side (Sartor-Glittenberg and Powers, 1993; Vallar et al., 1993a, 1995). Patients show disastrous constraints in everyday life as in safety, postural stability and motor functions (Carey, 1995; Carey et al., 1996; Dijkerman and De Haan, 2007) and impaired arm position sense (further termed APS) is associated with poorer and longer motor recovery of the hemiparetic or hemiplegic arm (Kuffosky et al., 1982; Wade et al., 1983; De Weerdt et al., 1987; Wadell et al., 1987; Feys et al., 2000). Historically, impaired APS has been considered to have the same incidence after lesions to the right and left hemisphere (Shah, 1978; Vallar et al., 2003; Reinhart et al., 2012). In contrast, recent studies found a strong relationship between APS disorders, lesions to the right hemisphere and left spatial neglect (Pizzamiglio et al., 1990; Vallar et al., 1993a, 1995; Schmidt et al., 2013a), and that impaired APS occurs more often after right- than left-hemisphere lesions (Sterzi et al., 1993). Moreover, there is also a higher incidence of left spatial neglect following right-brain lesions vs. right neglect after left-hemisphere lesions (Azouvi et al., 2002; Beis et al., 2004; Kleinman et al., 2007; Gottesman et al., 2008; Husain, 2008; Suchan et al., 2012). These convergent findings suggest a common underlying mechanism resulting in a higher incidence of both disorders in patients with right-hemisphere lesions. Another argument in favor of such a common mechanism is the observation that “bottom-up” treatments of the neglect syndrome based on sensory stimulation such as optokinetic or vestibular stimulation not only improve *visuospatial* neglect symptoms (optokinetic: Pizzamiglio et al., 1990; Kerkhoff et al., 2006, 2013, in press; vestibular: Vallar et al., 1993b), but also temporarily reduce the disordered APS in left neglect (optokinetic: Vallar et al., 1993a, 1995 vestibular: Rode et al., 1992; Rode and Perenin, 1994; Schmidt et al., 2013a). This finding favors the assumption that position sense deficits in spatial neglect have a non-visual component. This entails the defective perception of the spatial position of body parts (i.e., the contralesional, left arm) due to neglect and an ipsilesional shift in the spatial representation of these body parts and external objects in space. This model of bodily perception (Vallar et al., 1993a) was used to explain the above-mentioned findings in patients with neglect. According to this model, incoming sensory (e.g., proprioceptive) information from each side of the body is first processed by each hemisphere, but with a stronger contralateral processing stream. Second, somatotopic representations are entered in an egocentric representation of the body. Interestingly, there is an interhemispheric imbalance with a greater ipsilateral body representation of the right body side and a smaller representation of the left body side. This results in a higher susceptibility of the right cerebral hemisphere for left-sided body-related deficits when lesioned. In summary, in patients with right-hemisphere lesions the building-up and updating of the egocentric representation of their body and of the extrapersonal space is perturbed (Vallar et al., 1993a). Due to the assumptions about an asymmetric body representation, this model also explains the asymmetric incidence and why neglect and impaired APS so often are jointly impaired after right-brain lesions. Moreover, a right-hemisphere dominance in spatial perception, e.g. of limb movements, is also supported by imaging studies (e.g., Naito et al., 2005).

### Assessments of position sense

To date, most studies in clinical and healthy populations have examined lower limb position sense, although several studies have also measured position sense of the upper limb (e.g., Vallar et al., 1993a, 1995; Carey et al., 1993; Schmidt et al., 2013a). Assessment of limb position sense is often conducted by passively moving a single joint to a requested position in the horizontal or vertical plane, while other paradigms require the subject to actively move the limbs toward a target position (Jones et al., 2010). Such experiments investigate the accuracy of “active” limb positioning toward a requested target position, typically in the absence of vision. There are two common paradigms for “active” position sense tasks which vary in their demands on cognitive processing: in “ipsilateral remembered matching tasks” the subject's hand is guided by the examiner to a target position (Goble et al., 2009a). After returning the hand to the starting position, the subject is required to reproduce the target position with the same forearm only supported by his/her proprioceptive memory. These tasks make predominantly demands on the retrieval of memory-based proprioceptive information. In “contralateral concurrent matching tasks” the hand is positioned in a target position but is not being returned to the starting point. Instead, subjects are asked to match the target position with the other hand (Goble et al., 2009a). This kind of tasks requires the interhemispheric transfer of proprioceptive information. “Contralateral remembered matching task” are a combination of the two former ones in which the forearm is returned to the start position and the target position has to be reproduced by matching with the other forearm (Adamo et al., 2007). This latter task poses the highest demands on cognitive abilities during proprioceptive testing, which in turn can influence the former ones, particularly in the elderly (Li and Lindenberger, 2002). Apart from this, those more sophisticated methods also require some basic cognitive capacities (i.e., working memory, cf. Adamo et al., 2007, understanding complex instructions) which are often impaired after acquired brain damage. Evidently, such testing protocols of position sense require different sensory-motor and of course neural mechanisms and assessment methods must take into account the motor and cognitive impairments of patients after stroke (Carey et al., 1996). As a consequence, clinically suitable assessment methods should ideally be simple enough to be applicable in most patients or healthy subjects (Carey et al., 1996), and at the same time sensitive enough to detect even subtle impairments in a limited time.

Most studies of position sense, so far, used paradigms like pointing (Vallar et al., 1993a), reaching (Gordon et al., 1995), matching (Van Beers et al., 1998; Newport et al., 2001) or other judgment tasks (Wilson et al., 2010) to analyze APS in patients and healthy subjects. These methods provide most often ordinal or categorical ratings. Some of them only use a three—(Sterzi et al., 1993) or four-point scale (Vallar et al., 1993a, 1995) which are discrete scales and deliver only qualitative scores which are often not sensitive to changes after modulation or therapy and are not able to discriminate subtle deficits (Dukelow et al., 2010). Furthermore, some methods lack age—and sex-specific normative data and/or psychometric criteria (i.e., objectivity, reliability; Carey, 1995). Clinicians assess position sense often merely by asking patients to discriminate whether their finger or toe is moved upward or downward by the experimenter (Sterzi et al., 1993; Bickley, 2012), finger finding, positional mimicry or two-point discrimination (Lincoln et al., 1991) as well as by using the thumb localizing task (Hirayama et al., 1999). These clinical assessments also show no or poor psychometric criteria (e.g., interrater reliability), are also not sensitive enough and lack normative data (Garraway et al., 1976; Lincoln et al., 1991; Carey, 1995). However, recent studies show that there are some promising tools available for the quantitative evaluation of sensorimotor functions of upper extremities. For example, robotic devices circumvent the above-mentioned limitations of standard clinical assessment scales (for a review, see Scott and Dukelow, 2011). In this context the bilateral robotic exoskeleton called KINARM (Scott, 1999) has to be mentioned that measures horizontal limb position sense e.g. in mirror matching (Dukelow et al., 2010; Fuentes and Bastian, 2010) or reaching tasks (Coderre et al., 2010). Another new method for assessing hand position sense uses a magnetic motion tracking system with sensors attached on each hand in order to record movement trajectories in 3D coordinates (Leibowitz et al., 2008). While such sophisticated methods undoubtedly reveal interesting and novel scientific insights into the spatial and kinematic aspects of proprioceptive tasks, they may also show limitations in their clinical suitability. Hence, such robotic devices may entail the risk of automatic movements without control for patients with motor impairments and reduced flexibility in limbs. Moreover, they are often too complex for everyday clinical practice. Therefore, assessment tools for limb position sense which are easy-to-use, quick to perform, sensitive to changes (i.e., due to therapy) and which have normative values are needed. Carey et al. (1996; 2002 for more details) developed such a quantitative measure, termed the Wrist Position Sense Test that meets these afore-mentioned criteria. We adopted their approach to develop a similar test of APS in the horizontal plane, with emphasis on the static, endpoint component of proprioception.

The aims of the present study are threefold: First, we shortly describe this recently developed device for the assessment of horizontal APS of both forearms. Note that we have deliberately chosen a simple and easy-to-use device that is suitable for acute stroke patients, can be performed quickly within the limited time available in the clinical context, and is sensitive enough to detect changes throughout modulation or therapy. Second, we report normative data from 87 healthy subjects in the age range of 20–77 years for both arms and sexes collected with this new device. Third, we analyzed possible laterality, age or sex effects of APS for both arms, as this might offer interesting insights into the hemispheric (a)symmetry of position sense and can be related to impairments of APS in stroke patients. Finally, we discuss our results in relation to clinical findings of disturbed body cognition and awareness (i.e., somatoparaphrenia) in stroke patients and other (pre-)clinical disorders showing disturbed processing or awareness of their own body.

## Methods

### Participants

A total of 87 healthy subjects participated in the present study. They were recruited by public bulletins on campus, circular emails and by word of mouth. Inclusion criteria were right-handedness according to the forced-choice hand preference questionnaire by Annett (Salmaso and Longoni, 1985) and visual acuity of at least 0.5 (20/40 snellen equivalent) for the near viewing distance (0.4 m) in order to see the red LED, with all subjects wearing corrective glasses if necessary. Moreover, grip force was measured by Jamar hand grip dynamometer (Degasport, D-83115 Neubeuern, Germany) to rule out a potential influence of hand strength on APS. Participants had to hold their shoulder in 90° of abduction and their elbow in 30° of flexion by the side of their body, grip strength was measured in three trials for each hand and the outcome parameter was the averaged strength in kilogram, separately for each arm (see also Schmidt et al., 2013c). Exclusion criteria were a history of neurological and psychiatric disorders, dementia and physiological impairments of the arm which would not allow arm movements (see Table 1). Subjects were divided into three age groups: 20–40 years old (*n* = 40; mean age = 27.8; range = 20–40 years; 12 males, young group), 41–60 years old (*n* = 22; mean age = 52.9; range = 44–58 years; 11 males, mid-aged) and > 60 years old (*n* = 24; mean age = 67.8; range = 61–77 years; 13 males, old group). The three groups did not differ significantly with respect to sex [χ^2^(df = 2) = 3.97, *p* = 0.137]. All subjects had at least 1.0 (20/20 snellen equivalent) visual acuity for the near viewing distance and all gave their written consent prior to the examination in accordance with the Declaration of Helsinki II and the local ethical guidelines (Ärztekammer Saarland, Germany).

**Table 1:** **Demographical and experimental data of the three age groups and total group**.

**Age group**	***N***	**Mean age (range)**	**Sex**	**Handedness/Mean laterality quotient (range)**	**Mean visual acuity near (range)**	**Mean grip force, right arm (range)**	**Mean grip force, left arm (range)**
1 (20–40 years)	40	27.8 (20–40)	12 m, 28f	94.13 (40–100)	1.1 (0.8–1.25)	31.2 (16–57)	28.9 (13–57)
2 (41–60 years)	22	52.9 (44–58)	11 m, 11f	94.86 (25–100)	1.0 (0.8–1.25)	32.1 (20–63)	30.0 (16–52)
3 (>60 years)	25	67.8 (61–77)	13 m, 12f	97.60 (40–100)	1.0 (0.8–1.25)	31.1 (10–55)	28.5 (12–49)
Total	87	49.5	36 m, 51f	95.31	1.0	31.4	29.1

Abbreviations: m, male; f, female; handedness/lateralization quotient: +100: extreme right-handedness, 0: ambidexter, −100: extreme left-handedness; visual acuity: binocular, decimal letter acuity for the near viewing distance (0.4 m; 1.0 = 100% = 20/20 Snellen equivalent); grip force in kg (see text for further details).

### Arm position device

**Figure 1:**
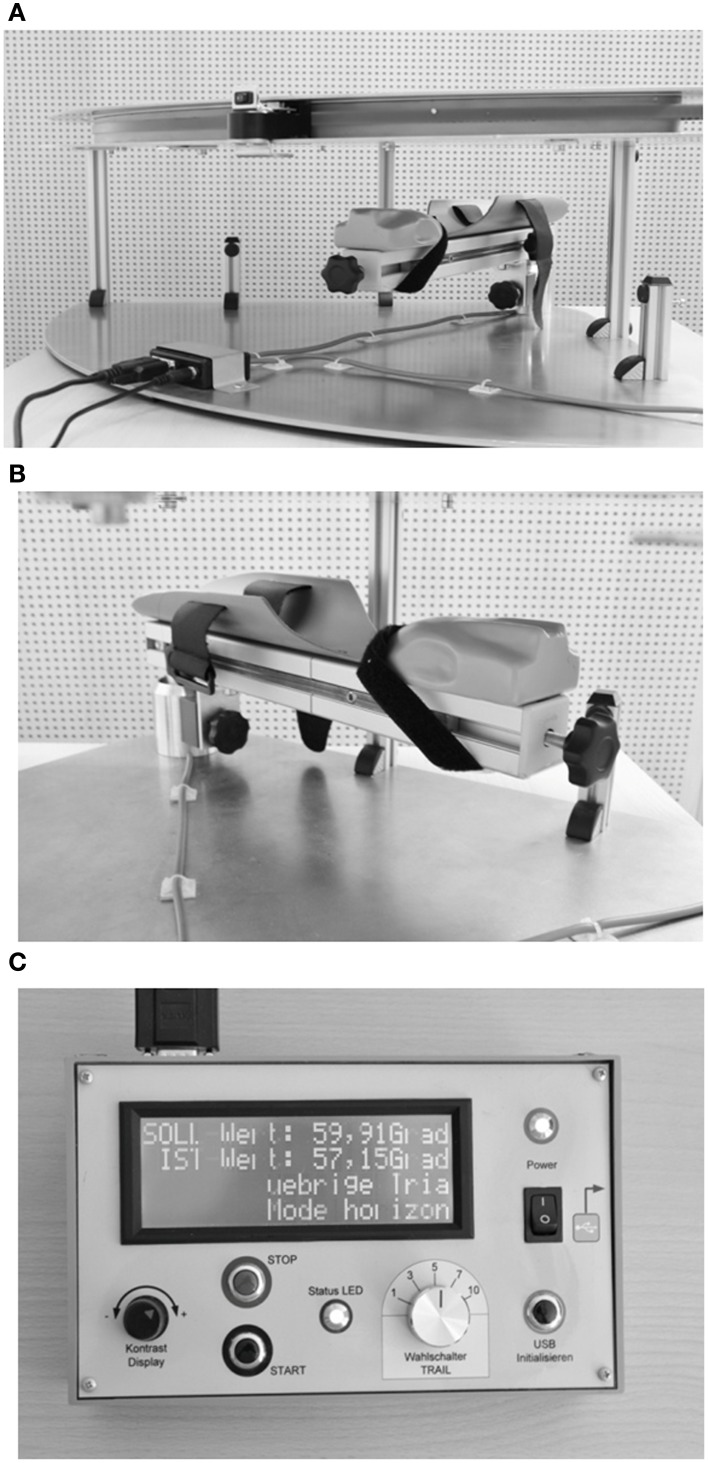
**(A)** Layout of the arm position device (APD, see text for further details). **(B)** Arm support. **(C)** Control panel.

We developed an opto-electronic device (“Arm position device”, APD, Figures 1A–C) specifically for measuring angular forearm displacement of the elbow joint in the horizontal plane. The APD consists of a 0–90° circuit with a small red LED lamp attached to the circuit. The LED lamp was manually adjusted by the examiner to set the optically required target position. The subject's forearm was placed and fixed with palm down on an arm support with the index finger lying extended on a special gap while the other fingers form a fist. This made sure that the tip of the index finger of all subjects (irrespective of individual arm length) was always positioned in the same spatial position relative to the LED. The circuit of the gap on the arm support and the circuit of the LED were perfectly aligned above each other and, therefore, the forearm was not visible for the patient (see Figure 1). The arm support was likewise moveable in the horizontal plane from 0–90° for elbow joint rotation in order to locate individual's actually horizontal arm position. A digital control panel showed the difference in degrees of visual angle between the optically required position (position of the LED) and the current position of the subject's forearm (position of the arm support) with a resolution of 0.1° [for further details, see Schmidt et al. (2013a,c)].

### Procedure

**Figure 2:**
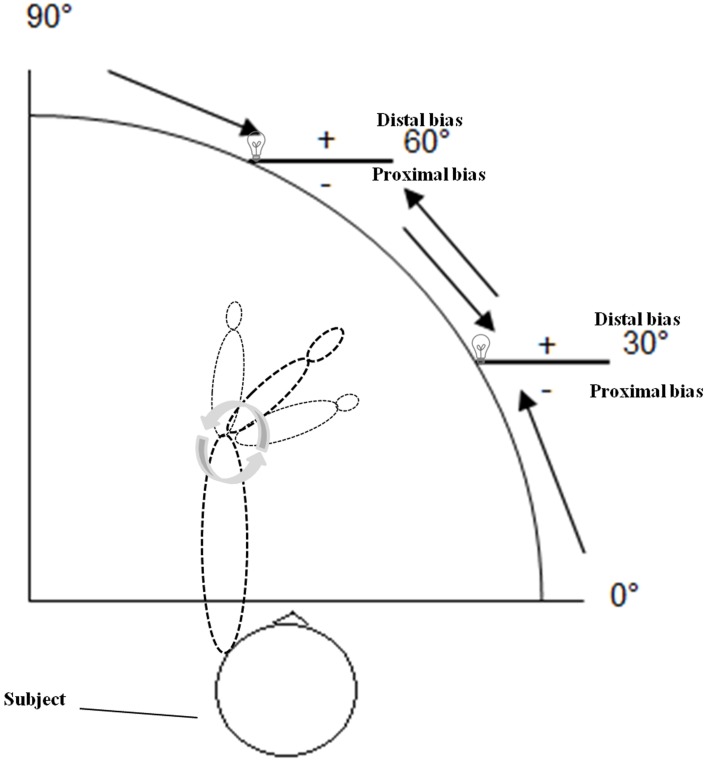
**Schematic drawing of the arm position device (APD) used in the present experiment when the left forearm was tested.** Two different angle positions of the elbow joint (30 and 60° flexion) from four different starting positions: 90° (forearm completely extended), 60° flexion, 30° flexion, 0° (forearm completely bent) were tested. Participants performed six trials per LED position, that is three trials +30° (bent movements) and three trials −30° (extended movements) of the true target position (see direction of the arrows). Negative symbols indicate proximal deviations (toward the own body), positive symbols distal deviations (away from the own body) (see text for further details).

During assessment of APS subjects were sitting upright in front of the APD, with either the right or the left arm resting on the arm support (Figure 1). The sequence of the arms tested was pseudo-randomized across subjects. The examiner, sitting in front of the subject, moved the subject's forearm toward the intended position (indicated by the LED lamp) by shifting the arm support with an attached knob in order to enable a constant velocity (average: 4.3 deg/s). Subjects did not actively move their arm to the required position but rather their arm was moved passively by the examiner. Participants were asked to verbally specify the point when their index finger was exactly below the LED lamp. The center of rotation of the device was the elbow joint. The experimenter moved the forearm to two different angle positions of the elbow joint (30° and 60° flexion) from four different starting positions: 90° (forearm completely extended), 60° flexion, 30° flexion, 0° (forearm completely bent), resulting in 30° movements per trial, respectively (Figure 2). Participants performed six trials per LED position, that is three trials + 30° (bent movements) and three trials −30° (extended movements) of the true target position (see direction of the black arrows in Figure 2), resulting in 12 trials per arm, which were performed in random order for controlling of sequence effects. Trials were averaged for the analyses, separately for each arm. During measurement the room was darkened (1 Lux illumination) and participants wore a black cape that ensured that the arm that was tested (up to the shoulder) was occluded from vision. Thus, visual cues (i.e., of the arm or the environment) were not available for the subject except the red LED. Participants did not receive any feedback about their performance, nor were there any time constraints for performing the task. Each test session were performed within a maximum of 20 min for both arms.

### Statistics

Due to the focus on static position sense, rather than kinesthetic movement sense, the final scores were mean deviations of the subject's actual horizontal arm position from the required LED position (in °), separately for each arm.

First, we calculated the absolute errors (AE), the mean unsigned deviations in degrees from the required target position, irrespective of their direction. Second, the constant errors (CE) were computed as the mean signed deviations from the target position in degrees. Positive values indicate a distal bias (away from the own body), negative values a proximal bias (toward the own body; see positive and negative symbols in Figure 2). This parameter serves as an indicator of accuracy in APS. Third, the interval of uncertainty was determined by subtracting the minimal from the maximal absolute deviation of the 12 trials. This parameter indicates the complete range within which the subject considers the index finger as exactly below the LED lamp. This value was used to calculate the difference threshold or difference limen (DL), defined as one-half of the interval of uncertainty and, thus, serves as an indicator of stability and precision in APS. Analyses of variance with the between factors “age group” (20–40 years, 41–60 years, >60 years) and “sex” (male, female) and the within factor “arm” (right, left) were carried out, separately for AE, CE and DL with subsequent Bonferroni-adjusted *t*-tests for multiple comparisons (Holm, 1979). One sample *t*-tests against zero were run for the CE of each age group, separately for the left and right arm. In addition, two tailed Spearman correlation coefficients were computed for the three outcome parameters between the right and the left arm as well as between grip strength and CE. The alpha-level was set at *p* = 0.05, two-tailed for all analyses.

## Results

Table 2 summarizes the mean results for the AE, CE, and DL in APS measured with the APD for both arms and for the three age groups separately. In addition, 95% confidence intervals are indicated, that may be helpful for clinical use.

**Table 2:** **Summary of the normative data for APS measured with the APD for both arms, separately for the three age groups and the three outcome parameters: mean absolute errors (AE), mean constant errors (CE) and mean difference threshold (difference limen, DL) in degrees**.

	**20–40 years**			**41–60 years**			**>60 years**		
	**AE**	**CE**	**DL**	**AE**	**CE**	**DL**	**AE**	**CE**	**DL**
**Right arm**									
Mean (°)	4.68	−0.57	0.21	4.65	−2.55	0.25	4.99	−2.00	0.54
*SD* (°)	1.78	3.71	1.34	1.77	3.31	1.17	2.99	3.40	2.69
Confidence intervals (°)	4.11–5.25	−0.1,76–0.62	2.66–3.52	3.86-5.44	−4.02–(−1.08)	2.26–3.30	3.77-6.23	−3.41-(-0.60)	2.16–4.38
**Left arm**									
Mean (°)	4.28	−0.89	.24	3.89	−1.81	.32	4.23	−1.14	0.23
*SD* (°)	1.76	3.36	1.54	1.72	3.15	1.51	1.68	2.79	1.13
Confidence intervals (°)	3.71–4.84	−1.97–0.18	2.20–3.19	3.14–4.66	−3.20–(−0.41)	1.95–3.29	3.54–4.93	−2.29–0.01	1.98–2.91

Ninety-five percent-confidence intervals are indicated. SD, standard deviation.

### Absolute errors

**Figure 3:**
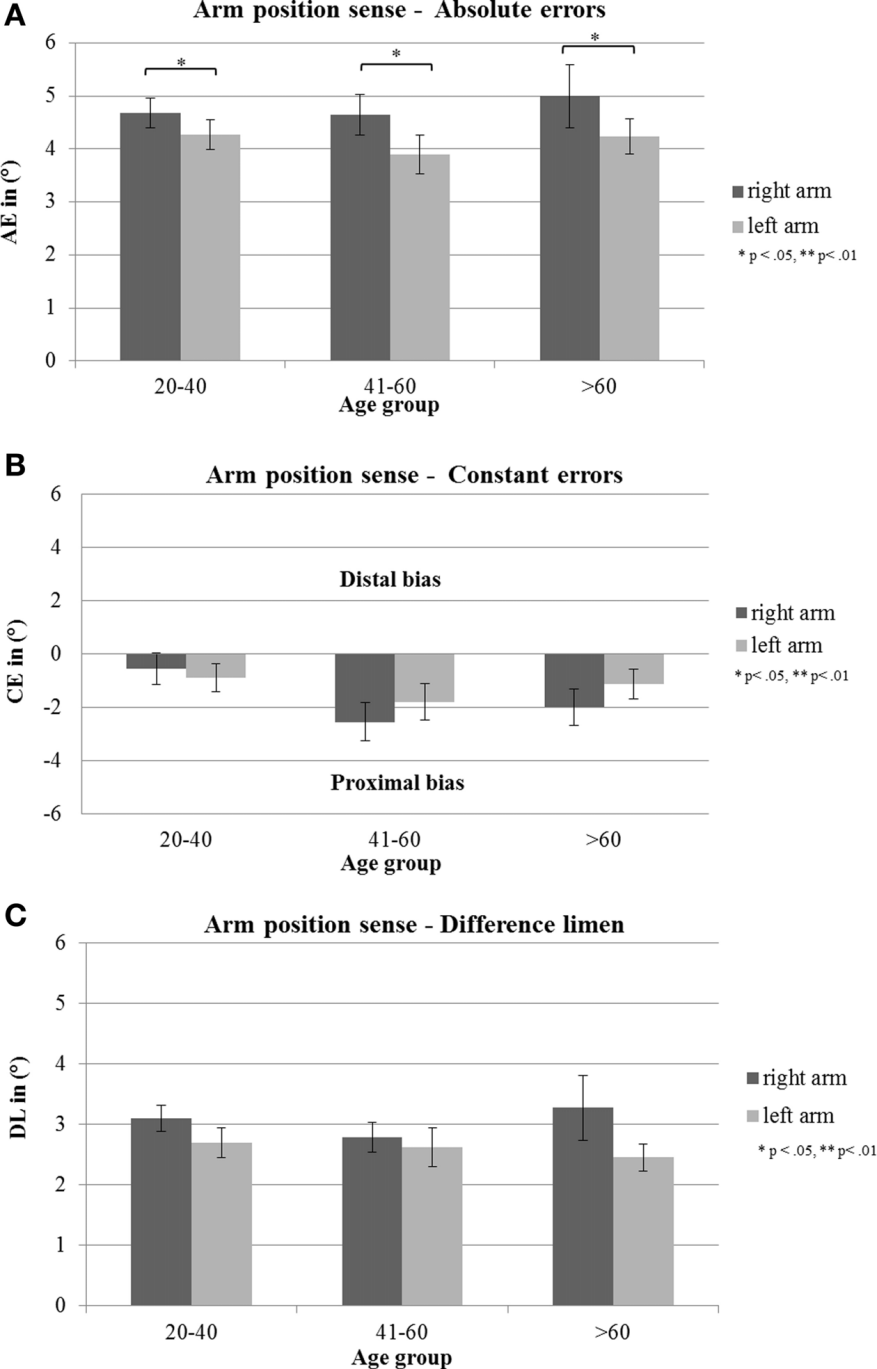
**(A)** Mean absolute errors (AE; in degrees), **(B)** mean constant errors (CE; in degrees) and **(C)** mean difference threshold (difference limen, DL, in degrees) with standard error of the mean (s.e.m) of the three age groups of right and left arm. Negative errors indicate proximal deviations (toward the own body), positive errors distal deviations (away from the own body).

There was a significant main effect for arm [*F*_(1, 81)_ = 4.02, *p* = 0.048, η^2^ = 0.042]. Subsequent *t*-tests showed that AE in the right arm were significantly larger than in the left arm [*T*_(86)_ = 2.18, *p* = 0.032]. The analyses showed no further significant main effect of age group [*F*_(2, 81)_ = 0.32, *p* = 0.725, η^2^ = 0.008] or sex [*F*_(2, 81)_ = 0.01, *p* = 0.929, η^2^ = 0.000]. Factors did not interact significantly with each other [arm × age group: *F*_(2, 81)_ = 0.33, *p* = 0.724, η^2^ = 0.008; arm × sex: *F*_(1, 81)_ = 0.18, *p* = 0.670, η^2^ = 0.002; age group × sex: *F*_(2, 81)_ = 0.89, p = 0.413, η^2^ =0.022; arm × age group × sex: *F*_(2, 81)_ = 0.89, *p* = 0.415, η^2^ = 0.021] (see Figure 3A).

### Constant errors

The analyses showed no significant main effect of age group [*F*_(2, 81)_ = 2.86, *p* = 0.063, η^2^ = 0.066], sex [*F*_(1, 81)_ = 1.61, *p* = 0.208, η^2^ = 0.019] or arm [*F*_(1, 81)_ = 0.93, *p* = 0.337, η^2^ = 0.011]. Factors did not interact significantly with each other [arm × age group: *F*_(2, 81)_ = 0.65, *p* = 0.524, η^2^ = 0.016; arm × sex: *F*_(1, 81)_ = 0.04, *p* = 0.853, η^2^ = 0.000; age group × sex: *F*_(2, 81)_ = 0.87, *p* = 0.423, η^2^ = 0.021; arm × sex × age group: *F*_(2, 81)_ = 0.02, *p* = 0.985, η^2^ = 0.000] (see Figure 3B).

### Difference limen

There was no significant main effect of age group [*F*_(2, 81)_ = 0.18, *p* = 0.831, η^2^ = 0.004], sex [*F*_(1, 81)_ = 0.47, *p* = 0.495, η^2^ = 0.006] or arm [*F*_(1, 81)_ = 3.22, *p* = 0.077, η^2^ = 0.038], as well as no interaction effect [arm × age group: *F*_(2, 81)_ = 0.575, *p* = 0.565, η^2^ = 0.014; arm × sex: *F*_(1, 81)_ = 0.11, *p* = 0.741, η^2^ = 0.001; age group × sex: *F*_(2, 81)_ = 0.09, *p* = 0.909, η^2^ =0.002; arm × sex × age group: *F*_(2, 81)_ = 0.18, *p* = 0.837, η^2^ = 0.004] (see Figure 3C).

### Additional analyses

There were significant correlations (Spearman correlation coefficients) between the left and right arm, respectively AE [*r*_s_ = 0.29; *p* = 0.006], CE [*r*_s_ = 0.27, *p* = 0.01], and DL [*r*_s_ = 0.21, *p* = 0.049]. There were no significant correlations between grip strength and CE of the right arm [*r*_s_ = 0.084, *p* = 0.453] or the left arm [*r*_s_ = − 0.019, *p* = 0.867], suggesting that APS is independent of grip force.

One sample *t*-tests against zero revealed that the CE in the age group of 41–60 years differed significantly from zero for the right [*T*_(21)_ = −3.61, *p* = 0.002, *d* = − 2.55] and for the left arm [*T*_(21)_ = − 2.69, *p* = 0.014, *d* = −1.80]. In the age group >60 years there was a significant difference from zero for the CE in the right arm [*T*_(24)_ = −2.95, *p* = 0.007, *d* = −2.00] but not in the left arm [*T*_(24)_ = −2.05, *p* = 0.051, *d* = −1–14]. Hence deviations were into the *proximal* direction toward the own body in both arms for these two age groups. CE in the age group 21–40 did not differ significantly from zero neither for the right arm [*T*_(39)_ = −0.97, *p* = 0.337, *d* = −0.57] nor for the left arm [*T*_(39)_ = −1.69, *p* = 0.099, *d* = −0.90].

## Discussion

The following results were found: (i) Healthy subjects show deviations in APS in both arms, especially in AE. (ii) There is no decrease in APS accuracy or precision with age. (iii) No significant sex differences in APS performance were found. (iv) AE were significantly higher in the dominant right forearm as compared to the non-dominant left arm in the right-handed participants. We will discuss these aspects consecutively below.

### Arm position sense in healthy subjects

#### Normative data

The present study reports normative data of APS in the horizontal domain from a rather large group of healthy subjects up to an age of 77 years, assessed with a new device (APD), allowing to determine angular deviations with a resolution of 0.1°. We found slight deviations in APS from the visual reference in all three age groups of healthy subjects. These results are compatible with the findings reported by Fuentes and Bastian (2010) who also observed different variations in proprioception across space and task demands in healthy subjects. Such variant estimates of arm position—which often were toward the own body (hence proximal bias)—may represent a “safety mechanism” of the proprioceptive system to prevent injury to the limbs by shifting them toward the own body (Fuentes and Bastian, 2010).

The normative data obtained in our study could assist in providing information about the normal range in APS of healthy subjects of different ages. Moreover, they are suitable to track changes in the accuracy and precision of APS into the normal range of healthy subjects in patients due to therapeutic interventions, as e.g., recently demonstrated during and after galvanic vestibular stimulation (GVS) in patients with left spatial neglect following stroke (Schmidt et al., 2013a).

Apart from these clinical and practical aspects, our study contributes the surprising at first glance finding that healthy, dominant right-handers produced less AE in the horizontal APS of their non-dominant, left arm in the absence of vision than of their preferred right arm, although they did not show differences in accuracy. This capacity was obviously unrelated to hand preference and grip force values, which were uniformly higher in the right arm in our right-handed sample. This finding corroborates a very similar finding from a recent study showing that healthy right-handers are not better in horizontal APS in their dominant, right hand as compared to their non-dominant, left hand (Schmidt et al., 2013c). Recent studies revealed also that it depends on task demands which limb shows better performance in proprioceptive tasks (Goble et al., 2006; Goble and Brown, 2007, 2009). Interestingly, it is not always the dominant limb but rather the non-dominant, left limb in right-handers, especially in static, position-related proprioceptive sense (Goble and Brown, 2008), suggesting a dominance of preferred right arm/left hemisphere for motor action and a non-preferred left arm/right-hemisphere sensory dominance for using proprioceptive feedback, especially in the absence of vision (Goble and Brown, 2008). The higher precision of APS in the left arm of healthy right-handers in the present study most likely reflects a superior hemispheric capacity of the healthy right hemisphere in this proprioceptive-spatial task. This result indicates a clear right-hemisphere superiority for left APS in right-handers—at least for static endpoint position sense in the horizontal plane—and neatly fits to the more frequent and more severe deficits in APS for the left arm in stroke patients with left spatial neglect due to right-hemisphere brain lesions (Vallar et al., 1993a, 1995; Schmidt et al., 2013a).

#### Right- vs. left-handers

This asymmetry appears to be selective for right-handers, but not for left-handers (Schmidt et al., 2013c) and does not confirm the inverse asymmetry found for left-handers in proprioceptive target matching tasks (Goble et al., 2009b). In this recent study, right-handers showed a significant direction-specific bias in both forearms in APS, whereas left-handers did not have a significant deviation in *any* arm. Furthermore, GVS temporarily disrupted this proprioceptive ability of the left arm in dextrals but had no effect in the matched sinistrals. These findings point, first, to superior arm proprioception in left-handers for *both* arms and, second, to a greater susceptibility of the systems involved in the building-up and updating of cortical body representations by incoming sensory (vestibular) information in right-handers. This, in turn, is compatible with a right-hemisphere dominance for vestibular functions in right-handers, because this unilateral, predominantly right-hemisphere, vestibular cortical representation is easier to disturb by vestibular stimulation, and a differential, probably more bilateral vestibular organization in left-handers, that more easily compensates for such disturbing effects of vestibular stimulation on APS (Schmidt et al., 2013c). Recent studies neatly fit to this general picture showing that left-handers perform better in body-related cognition tasks, both on the behavioral (e.g., Laeng and Peters, 1995; Linkenauger et al., 2009; Hach and Schütz-Bosbach, 2010) as well as on the neuroanatomical level (e.g., Amunts et al., 1996; Vingerhoets et al., 2012).

#### Proximal vs. distal biases

In order to analyze APS more precisely and to detect small deviations in APS in healthy subjects, we computed different types of errors (see Table 2). Concerning non-directional AE our healthy individuals showed deviations in APS which were, surprisingly, independent of age: right arm: 4.65°–4.99°, left arm: 3.89°–4.28°. Regarding signed errors, the finding that the mid-aged group showed significant deviations for both arms and the oldest age group in the right arm, hence in both cases toward the own body (proximal errors), suggest that these two age groups relied more on their own body as a reference frame for position sense as compared to the youngest age group. This could be interpreted as a slight age effect. However, a closer look at the data (see Figure 3B) shows that all age groups manifested a proximal bias in APS toward their body for both arms: right arm: −2.55° – (−0.57°), left arm: −0.89°− (−1.81°). This finding corresponds neatly with findings of recent studies concerning APS in healthy right-handers (Schmidt et al., 2013c) as well as in stroke patients with left neglect (Vallar et al., 1993a; Schmidt et al., 2013a). Apparently, the own body plays an important role for determining the position of own limbs in the absence of vision in the personal near space. This proximal error in APS might be interpreted as a kind of “productive” abnormality which is due to the importance of our own body as a reference for updating the position of body segments in relation to it.

#### Age

The most common assessment of proprioception in the elderly is assessment of the static position of limb segments (Goble et al., 2009a). Interestingly, we did not find a significant decrease in APS accuracy and precision with age. This finding is at first glance at variance with almost all other studies conducted on this topic, which show a clear age effect on proprioceptive abilities (Adamo et al., 2007), and a significant deterioration of limb position sense with age (Stelmach and Sirigu, 1986; Meeuwsen et al., 1993; Adamo et al., 2007; for a review, see Goble et al. (2009a). Interestingly, this deterioration in the ability to sense the position of a body segment with age is typically found in studies which used limb matching tasks (using both arms), as mentioned in the introduction, but not in studies using proprioceptive matching tasks with visual reference points where subjects indicate the felt position of their limb relative to a visual marker (Cressman et al., 2010). Moreover, the extent to which limb position sense is influenced by aging depends on aspects like the tested joint/limb segment, active/passive task, kind of analyzed error [for a review, see Goble et al. (2009a)] or task goal (Jones et al., 2012). Moreover, reduced proprioceptive acuity may also reflect age-related changes in cognitive functions (i.e., decisional factors, working memory) due to demands of the assessment methods as mentioned above. To circumvent such potentially influential factors, the examination of APS with the APD was kept as short and simple as possible. Therefore, potential confounding factors such as decreased memory abilities or reduced sustained attention with age (Reuter-Lorenz and Sylvester, 2005), or age-related deteriorations in cognitive processing which influence sensorimotor functions (Li and Lindenberger, 2002) were minimized in our sample, in contrast to ipsilateral remembered matching tasks. The fact that assessing APS with the APD requires matching of each forearm's position in relation to a visual reference point in peripersonal space and not in relation to the other arm, avoids another confounding factor of contralateral concurrent matching tasks, namely the interhemispheric transfer of proprioceptive feedback due to the age-related degeneration of the corpus callosum (Salat et al., 2005; Ota et al., 2006). Adamo et al. (2007) examined age effects on position sense for the elbow under three matching conditions which varied in requirements of memory and interhemispheric transfer: ipsilateral remembered, contralateral concurrent, and contralateral remembered condition. They found a main effect of age on absolute matching errors with greatest errors in the most demanding condition, which required both memory and interhemispheric transfer, in the older age group, suggesting that these tasks require more than merely position sense. Moreover, recent studies did not find age effects in all kinds of analyzed errors of position sense of specific limbs, e.g., of the finger (Ferrell et al., 1992) or the foot (Meeuwsen et al., 1993), when constant, direction-specific errors were analyzed, in contrast to unsigned errors. Therefore, the missing age effect in the present study can be explained by interaction effects of computed outcome parameter and the analyzed limb. This is consistent with the result that younger and older aging people show similar proprioceptive acuity when APS is assessed in a visual-to- proprioceptive matching task, such as in the APD in the present study (Ferrell et al., 1992; Cressman et al., 2010), without requiring proprioceptive memory or interhemispheric transfer. This finding suggests that the extent of proprioceptive recalibration with visual reference markers is independent of age and remains largely constant throughout the lifespan. Another explanation for the lack of age effects in the present study as compared to previous studies could be the different task demands as proposed by Cressman et al. (2010). According to Fuentes and Bastian (2010), endpoint limb positions are more robust against deteriorations due to age than angle position information. They argue that due to the greater behavioral need to estimate limb positions than joint angles, the brain may immediately encode limb position from peripheral sensory signals as compared to joint angle estimates which have to be extracted from these representations.

Apart from task-specific effects, there is clear evidence for age-related changes in the neural basis of proprioceptive processes (e.g., Goble et al., 2012), namely degenerative and plastic-adaptive processes in the aging proprioceptive system, both in the central as well as in the peripheral nervous system and muscular system [Adamo et al., 2007; for a review, see Goble et al. (2009a)]. This raises the question why some studies did not find age effects in proprioceptive matching tasks. One potential explanation may be that older subjects can compensate for this decline, due to implicit learning mechanism throughout life. This question will be a major challenge for future studies and could also inform us how proprioceptive impairments can be prevented or treated by exploiting such compensatory mechanisms.

#### Sex

No sex-specific differences were found in APS performance in the present study. This negative finding contradicts the widely shared assumption that males have better spatial skills as compared to females (Voyer et al., 1995). However, previously performed studies have not yet been able to explain the sex-specific differences in these tasks and simply assumed sex as a causal factor. Contreras et al. (2012) have studied this issue using three different spatial matching tasks. They found that sex was not important for correctly solving these tasks, but rather a specific type of process that determined participants' efficiency in solving a spatial task.

Therefore, the magnitude of the advantage that males may have over females crucially also depends on the type of spatial task. Accordingly, limb position sense imposes demands on the proprioceptive system in the personal space and might require the same underlying cognitive abilities in males and females and activate the same type of processes for solving the task in both sexes. This may explain the lack of any sex effects in our APS task.

### Clinical issues

Impaired limb position sense is a frequent and debilitating sequel after stroke (Shah, 1978; Smith et al., 1983; Carey, 1995). Positions sense disorders are likely to be caused by a failure to link somatosensory with egocentric information (Vallar et al., 1993a). Patients show constraints in performing activities of daily living, have problems in safety, postural stability and motor functions (Carey, 1995; Carey et al., 1996; Dijkerman and De Haan, 2007). In the clinic, patients with impaired APS show poorer and longer motor recovery of the hemiparetic or hemiplegic arm (Kuffosky et al., 1982; Wade et al., 1983; De Weerdt et al., 1987; Wadell et al., 1987; Feys et al., 2000).

Previous studies found that left-sided visuospatial neglect after right-brain damage is functionally associated with impaired arm position judgments in the contralesional arm (Schmidt et al., 2013a) and also with problems in other body-related spatial tasks such as left tactile extinction (Schmidt et al., 2013b) or in identifying left human hands (Reinhart et al., 2012). This proprioceptive deficit can be temporarily restored by GVS (Schmidt et al., 2013a). This improvement may be either due to a more veridical perception of their contralesional arm, or of the target LED, or of both components.

#### Relation to other body cognition disorders

The right-hemisphere superiority for proprioception in healthy right-handers - found in this but not in all other studies - and the greater left-sided impairments after right-hemisphere lesions are compatible with other “dysfunctions” of the right cerebral hemisphere that result in left-sided body-related disorders such as somatoparaphrenia (Gandola et al., 2012) or neuropsychiatric disorders without a history of brain damage such as xenomelia (Berti, 2013; Hilti et al., 2013). Xenomelic subjects, who desire the amputation of healthy limbs, show a reduced activation in the right superior parietal cortex during tactile stimulation of the affected leg (McGeoch et al., 2011). Hilti et al. (2013) studied the brain areas associated with xenomelia for the left arm in 13 patients and found predominantly right-hemisphere brain abnormalities resulting in the strong desire for amputation of left-sided limbs. These brain areas correspond neatly with those identified in patients with somatoparaphrenia, where the patient feels that a paralyzed limb does not belong to his body (Gandola et al., 2012; Hilti et al., 2013) and has a blurred distinction between corporeal and extracorporeal objects, that is often associated with left motor and somatosensory deficits and neglect (Ronchi and Vallar, 2010). In turn, this lesion pattern, involving a fronto-temporo-parietal network in the right cerebral hemisphere, is typically associated with spatial neglect, hemiplegia and anosognosia (Gandola et al., 2012), which confirms the importance of right hemisphere for productive behaviors in personal and near extrapersonal space.

The overestimation of body size in patients with anorexia nervosa is proposed to be, besides psycho-affective causes, the result of impaired neural mechanisms supporting body representation, comparable to patients following stroke. In an interesting, innovative study Nico et al. (2010) compared body knowledge in anorexics, healthy subjects and patients with lesions of either the left or right parietal lobe after stroke. They found that patients with anorexia nervosa and those with right parietal damage selectively underestimated the extent of their *left* body boundary in a similar way. This finding confirms the important role of the right parietal cortex in building-up and updating of the representation of the body and peripersonal space (Graziano and Gross, 1995), also in anorexics (Grunwald et al., 2002).

### Limitations

We have tested the APS using a visual reference (LED) while most of the other studies on arm proprioception use non-visual tasks. This may have led to different results.

As another limitation, we did not collect data of the vertical or sagittal dimension and, therefore, we are not able to make conclusions about the generalization of our results on the entire egocentric coordinate system, as suggested in other studies (e.g., Vallar et al., 1995).

Moreover, we cannot exclude the possibility that we may have missed age-related deteriorations in proprioception in the form of an increased variability as a result of the limited number of trials in our assessment with the APD, as found with other devices and more trials in elderly subjects (Cressman et al., 2010).

## Conclusion

In summary, this study provides normative data from healthy subjects for APS of a wide age range (20–77 years) for both arms in the horizontal plane. APS in our healthy subjects was not significantly influenced by age or sex, but all right-handed healthy subjects showed significantly more accurate performance in their non-dominant (left) arm. This indicates a clear right-hemisphere superiority for left APS in right-handers. This finding neatly fits to the more frequent and more severe left-sided, body-related deficits in personal/peripersonal space tasks observed in patients suffering from right-hemisphere stroke (e.g., anosognosia for left hemiparesis, somatoparaphrenia, body neglect) as well as to the right-hemisphere abnormalities reported in some neuropsychiatric conditions associated with body perception or body representation deficits (e.g., anorexics).

### Conflict of interest statement

The authors declare that the research was conducted in the absence of any commercial or financial relationships that could be construed as a potential conflict of interest.

## Abbreviations

APS: arm position sense
APD: arm position device
AE: absolute errors
CE: constant errors
DL: difference limen
GVS: galvanic vestibular stimulation.
